# The Future of Vaccination

**Published:** 1896-05-16

**Authors:** 


### The Hospital

An Institutional Journal of

**The Medical Sciences and Hospital Administration,**

with

**AN EXTRA NURSING SUPPLEMENT.**

Vol. XX.—No. 503.] [May 16, 1896.

### The Future of Vaccination.

Interesting as is the history of vaccination, it is a matter of still greater practical moment to look forward, and in the light of what is past to forecast the future of this method of prophylaxis. It is one of the most striking proofs of Jenner's genius that his great invention was born before due time, and was so far out of touch with the knowledge of the period in which he lived that for nearly a hundred years it remained an isolated fact, unexplained by, if not totally at variance with, all that was known of the pathology of disease. It has only been within the last few years that the protective power of vaccination has been explained, and has been shown to be analogous with other facts in pathology.

Strongly as it has been asserted that vaccinia is a specific disease of the cow, modern investigations have shown not only that Jenner's original idea that it was small-pox modified by passing through the cow was correct, but that the protection obtained by such a modified virus is in full accord with that obtained in regard to other diseases by inoculation with their attenuated virus; so analogous, in fact, that these attenuations have come to be spoken of as vaccines.

It is not, then, altogether unnatural that people who are quite willing to submit to vaccination as a protection against such a loathsome disease as small-pox should be somewhat disturbed in mind at seeing how far the principle on which it is founded is capable of extension; for they ask themselves, whither does this tend, what is to be the end of it, and if we admit compulsory vaccination against small-pox, how can we prevent the enforcement of compulsory vaccination against all sorts of other diseases?

The answer to this question is simply this, that small-pox stands on a completely different platform from all other diseases. There is no other disease which is at the same time so infectious, so fatal, and so crippling as small-pox.

Although some process analagous to vaccination may be found useful as a protection against cholera, yellow fever, and malignant malaria in tropical areas, the question of protective vaccination does not arise in regard to any disease except small-pox in temperate climes.

Some people profess to believe that with the advancement of sanitation zymotic diseases will become things of the past. We wish that we could agree with such hopeful prognostications. The fact is, man and his diseases have grown up together, and every fact in natural history points to the probability that disease germs will accommodate themselves to any change in man much more readily than man will fit himself to new surroundings; and that, as regards small-pox, which affects the natural savage just as severely as the most civilised man, no conceivable improvement in physical condition will give man immunity.

To vaccination, then, we must have recourse, but not necessarily to the vaccination of to-day. So long as people choose to look on vaccination as a mere process which experience has taught to be prophylactic against small-pox, of course they must follow the receipt given them, and just as a cook puts certain ingredients into a pudding without knowing why, so they must go through the exact formula which Jenner found to be efficacious. Most unfortunately, the administration of the public vaccination of the people was years ago handed over to the Poor Law authorities and was fossilised under the dead hand of officialism which devoted itself to the enforcement of a certain historic form of pock, and thus while England is one of the worst vaccinated countries it also is one in which the vaccination raises the greatest outcry when compulsion is attempted. We still maintain the old creed that vaccination from arm to arm is the true way to safety, while other nations by animal vaccination practically disarm objections to the operation. The first step then in the future of vaccination must be the universal substitution of animal for arm to arm vaccination; the next the securing of aseptic vaccination, for there is but little doubt that as vaccination is too often done at present it is an inoculation with a very mixed infection. But we are far from saying that these steps should be the last. Everything points to the possibility of our being able before long to obtain pure cultures of the germs of small-pox as of other diseases entirely independent of any living media, and it is not unreasonable to believe that when that result shall be obtained vaccination will be a much less serious business than it is at present. Perhaps it is permissible to look ahead still further, and anticipate the time when even the inoculation of living germs, attenuated though they be, may be found to be unnecessary, and when vaccination, instead of being the production of a disease, will consist essentially in the administration of a chemical substance produced by cultivation of the germ, a drug in fact, which shall give immunity. This would, indeed, be a thing worth having. We now celebrate the centenary of a discovery which, while full of practical usefulness, has been of wondrously slow fruition in regard to science. Let us hope that in the century now opening progress may be of quicker growth.

